# A Gated Multiscale Multitask Learning Model Using Time-Frequency Representation for Health Assessment and Remaining Useful Life Prediction

**DOI:** 10.3390/s23041922

**Published:** 2023-02-08

**Authors:** Tong Wu, Tengpeng Chen

**Affiliations:** 1 Department of Instrument and Electrical Engineering, Xiamen University, Xiamen 361102, China; 2 Shenzhen Research Institute of Xiamen University, Shenzhen 518000, China

**Keywords:** health assessment, remaining useful life prediction, multi-task learning, multi-scale learning, time-frequency representation, mixture-of-experts

## Abstract

Health assessment and remaining useful life prediction are usually seen as separate tasks in industrial systems. Some multitask models use common features to handle these tasks synchronously, but they lack the usage of the representation in different scales and time-frequency domain. A lack of balance also exists among these scales. Therefore, a gated multiscale multitask learning model known as GMM-Net is proposed in this paper. By using the time-frequency representation, GMM-Net can obtain features of different scales via different kernels and compose the features by a gating network. A detailed loss function whose weight can be searched in a smaller scale is designed. The model is tested with different weights in the total loss function, and an optimal weight is found. Using this optimal weight, it is observed that the proposed method converges to a smaller loss and has a smaller model size than long short-term memory (LSTM) and gated recurrent unit (GRU) with less training time. The experiment results demonstrate the effectiveness of the proposed method.

## 1. Introduction

Roller bearings are frequently used in industrial systems due to its reliability which is seen as an element of safety in many electromechanical systems such as motors, aviation engines and wind turbines. Because of rigorous working conditions, bearing failure often happens, which can cause serious consequences such as sweep chamber and halt of the motors and wind turbines. As a result, the bearing health assessment and remaining useful life (RUL) prediction system is required to improve the reliability of the whole system via preventive maintenance [1].

Health assessment is a procedure of identifying the health state of the equipment as early as possible to get the information about the degradation. The RUL prediction is a procedure that estimates the remaining life of objects in the future and obtains the time before an object loses its operation ability. Health assessment is a task that is in some ways like fault diagnosis. Both of them are classification tasks which obtain the class to which one sample belongs. In the literature, most health assessment as a classification task consists of two key stages: feature extraction and health state recognition [2]. Typical methods in feature extraction include hidden Markov models [3], wavelet transform [4], and fuzzy formalisms [5], which are followed by artificial intelligence based methods such as support vector machine [6,7] and artificial neural network (ANN). Various neural networks have been proposed for a classification task, such as recurrent neural network (RNN), which includes long short-term memory (LSTM) and gated recurrent units (GRUs) [8], temporal convolutional networks (TCNs) [9], and deep belief networks (DBNs) [10]. However, these methods lack the ability to handle the data with variable lengths. Recently, technologies such as autoencoder provide an end-to-end method for fault diagnosis to handle inputs and outputs that are of variable-length sequences and decrease the time for the models to be trained [11,12]. The autoencoder architecture has been successfully applied to different areas. However, these methods cannot handle data from different sources from different sensors. Therefore, a modified deep autoencoder driven by multi-source parameters is proposed for fault transfer prognosis of aero-engine [13]. An imbalanced data classification method based on weakly supervised learning presented in [14] is utilized to make classifiers easier to learn an accurate decision boundary.

In practice, the safety of the whole industrial system cannot solely depend on health assessment because the degradation process of a roller bearing is continuous; it is too broad to just obtain a health state with discrete numbers. The remaining useful time is also required in order to determine whether maintenance is needed for extending the service life of the equipment and deciding whether to replace certain components. The RUL is estimated from condition data measured by different kinds of sensors placed on the component [15]. The RUL prediction method can be classified into three types, namely model-based, data-driven, and hybrid methods [16]. The model-based methods consist of developing physical and statistical models to represent the degradation process of a component, which are then used to estimate the RUL. Kalman filtering [17], particle filter [18], and Wiener process model [19] are some of the commonly used model-based models. However, one of the limitations for model-based methods is the uncertainty and simplifications of the prior knowledge the model with parameters needed to estimate. Therefore, data-driven methods are proposed to solve this problem by using the operational data that are monitored by sensors and are related to the health condition of the system. Similar to health assessment, machine learning has been widely employed in data-driven methods, such as support vector machine [20], ANN [21], and Bayesian networks [22]. The hybrid method takes advantage of the model-based method and data-driven method to simplify some of the computation [23]. However, due to the model complexity and difficulty in acquiring experimental test data, it is more challenging to predict the RUL than health assessment.

Both health assessment and RUL prediction can use the same input sampled by the sensors to achieve two tasks. Consequently, by leveraging on the advantages offered by both health assessment and RUL prediction, industrial applications can obtain great benefits from jointly using the two functions by sharing the same input. Health assessment can help evaluate the states of machines to obtain preventive maintenance in a timely manner, while RUL prediction can provide information on the remaining useful time for these machines to operate. The RUL prediction also needs health assessment to identify the health state so that machines can be more effectively maintained in order to decrease the downtime and increase economic benefits. In [24], the dual-task deep long short-term memory networks are proposed for joint learning of degradation assessment and RUL prediction of aeroengines. A joint-loss convolutional neural network architecture is proposed in [25] to capture common information of fault diagnosis and RUL prediction. However, the above methods do not use the different features in different scales, and they do not have the option to dynamically adjust the relationship between different features in different scales. In addition, a method to deal with information from different scales and different relative tasks is needed because one task or scale can affect the effectiveness of the other task of the model because they share the same parameters. Furthermore, the representation in time domain does not provide a simpler way to understand the degradation of the roller bearings; hence, another representation that is easier to implement is required. Therefore, we propose a neural network model that consists of multi-task learning, multiscale learning, time-frequency domain representation, and mixture of experts. The main contributions of this paper are as follows:

(1) By integrating the time-frequency domain representation with neural nets, the degradation features can be easily understood by both human and computers. The features of time-frequency representation in different scales can be obtained through multi-scale learning in the proposed method, which can make the system more intelligent to achieve the tasks;
(2) Health assessment and RUL prediction are considered as a single model in multitask learning, which makes the industrial measurement system simple to implement. These tasks are no longer viewed as individual tasks. Inspired by a mixture of experts, the proposed method uses a gating network in order to adapt to the difference among the different scales;
(3) The model with an optimal weight is found that the proposed method converges to a smaller loss and have a smaller model size than long short-term memory (LSTM) and gated recurrent unit (GRU) with less training time.

The rest of the article is organized as follows: Section 2 presents the key ideas of the proposed method. Section 3 describes the details of our proposed method which include the construction of the spectrogram and the GMM-Net. Section 4 discusses the experiment results and effectiveness of the proposed method by comparing with different gating nets. Section 5 concludes this paper.

## 2. Related Works

In this section, four related areas are discussed which include multi-task learning, time-frequency domain representation, multiscale learning, and mixture of experts.

### 2.1. Multi-Task Learning

Multi-task models can learn the similarities and differences between different kinds of tasks. Using such models can make each task more efficient and more accurate [26]. This is due to the five architectural mechanisms: the implicit data augmentation, attention focusing, eavesdropping, representation bias, and regularization. Multi-task learning can increase the sample size that are used for training by the different noise patterns of different tasks. It can also help the model focus its attention on the features which are important when additional information, which shows the relevance and irrelevance of different features, is added. Eavesdropping allows tasks to learn features that are difficult to learn. Representation bias can help the model to generalize in a similar environment with shared features. Finally, multi-task learning can reduce the risk of overfitting by acting as a regularizer.

**Figure 1:**
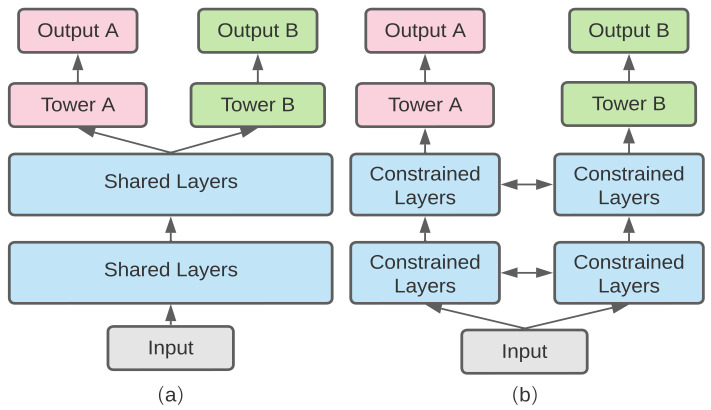
(**a**) Hard parameter sharing model; (**b**) soft parameter sharing model.

There are two representative models for multi-task learning in deep neural networks which are the hard parameter sharing and soft parameter sharing, as shown in Figure 1. The hard parameter sharing model is widely used. It is constructed by sharing the hidden layers between all tasks, while reserving some special output layers for different kinds of tasks, such as Latent Multi-Task Architecture Learning [27]. Conversely, soft parameter sharing is constructed using individual networks for each task while only making connections with some layers of different representations of different tasks such as Cross-Stitch Networks [28].

### 2.2. Time-Frequency Domain Representation

Signals have both time and frequency domain representations where each have their own advantages. In the time domain, signals can be easily represented as a function of time. However, most signals are difficult to analyze in time domain because these signals are not regular, and they cannot be represented in an easy manner to understand. Some examples of such signals are speech signals and noise signals. Hence, transformations are used to convert time domain representations into frequency domain representations in order to represent these signals because most signals contain only a few kinds of frequencies i.e., spectral sparseness. By combining time and frequency domain representations, more information can be extracted from raw signals. In reality, speech signals, music signals, and vibration signals can be represented using the time-frequency domain representations also known as spectrogram [29,30].

### 2.3. Multiscale Learning

Multiscale learning is frequently used in computer vision, natural language processing, and motion control [31]. Its working principle is inspired by the fact that people perceive various objects at different kinds of scales, where features from hierarchical representations of the object can be learned. For instance, the microscope can be considered as a composite system of cameras of different kinds of focal lengths. By using a combination of different cameras, the system can learn multiscale representations from the local scale to the overall scale. These systems similarly use different kernels that have different receptive fields to perceive the inputs including signals represented in both time and frequency domain.

### 2.4. Mixture of Experts

Different models are needed to deal with different problems. Since each model offers different benefits, an approach containing an ensemble of these different models are required. The ensemble of different models is proven to have the ability to improve the performance of the model [32]. A mixture-of-experts model can be used as a basic building block of deep learning systems. This block can select subnets, which are called experts, based on the inputs of the block by giving these subnets their weights, respectively. A mixture of experts can dynamically leverage source domains’ features to improve the model’s generalization capability.

## 3. Proposed Approach

**Figure 2:**
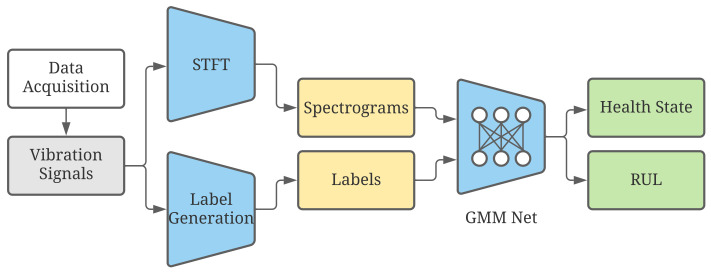
Framework of our proposed method for fault diagnosis and remaining useful life prediction.

After discussing the related works in the previous section, the framework of the proposed approach is shown in Figure 2. Firstly, the fast Fourier transformation is used to convert the raw data in time domain representation to frequency domain representation. Then, these two kinds of representations are combined together to time-frequency domain representation, where the data are divided into two kinds of data, i.e., training set and test set. By examining the features of raw data, each time-frequency domain representation can be given a label, which includes the state of health and the remaining useful life of the roller bearing. These new data can be used in time and frequency domain representation with their own labels to train the proposed neural network, which comprises three experts, gate network, and the tower network. The proposed method will be subsequently validated to check the correctness.

### 3.1. Calculation of Spectrogram

The spectrogram is a representation in time and frequency domain, which shows the spectrum of frequencies of a signal when it changes with time. It can be calculated from the raw signal in time domain by using the Fourier transformation. The sampled data in time domain can be divided into several segments, which are subsequently Fourier transformed to obtain the magnitude in the frequency spectrum. Every segment is represented as a vertical line in the spectrogram. In reality, this process is the computation of the squared magnitude of short-time Fourier transformation (STFT). In this paper, Fourier Transformation is sufficient to obtain the time-frequency domain representation of the vibration signal of roller bearings. For a signal in continuous time, the STFT is expressed as

(1) $$\begin{array}{c} \mathrm{STFT}\left(x\left(t\right)\right)\equiv X\left(\tau ,\omega \right)=\int_{-\infty }^{\infty }x\left(t\right)\omega \left(t-\tau \right)e^{-i\omega t}\;dt \end{array}$$

in which x(t) represents the original signal in time domain, and ω(t) refers to the window function that can cut out a segment from the original signal and perform the product of itself and the original signal. Since the signals used in this paper are the vibration signals with sampling, the following equation converts the signal from continuous time to discrete time and is expressed as

(2) $$\begin{array}{c} \mathrm{STFT}\left(x[n]\right)\equiv X\left(m,\omega \right)=\sum_{n=-\infty }^{\infty }x\left[n\right]\omega \left[n-m\right]e^{-j\omega n} \end{array}$$

with the signal x[n] and the window function ω[n]. The magnitude squared of the STFT is the spectrogram representation of the power spectral density of the signal and is expressed as

(3) $$\begin{array}{c} spectrogram\left(x[n]\right)={\left|X(m,\omega )\right|}^{2} \end{array}$$

The window function chosen is the Hann function, and it is expressed as

(4) $$\begin{array}{c} \omega \left[n\right]=sin^{2}\left(\frac{\pi n}{N}\right) \end{array}$$

which is a sequence of *N* + 1 samples. Because the length of the vibration signals is 2560, 256 is chosen as *N* to the length.

**Figure 3:**
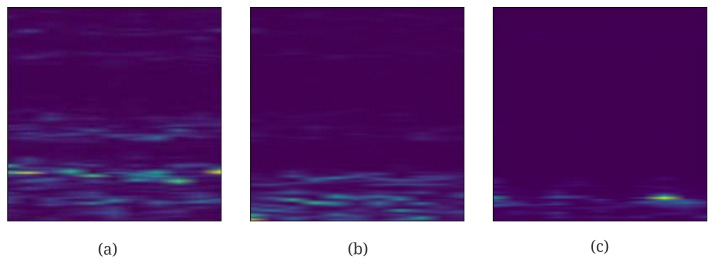
Spectrogram of vibration signals of bearings in different health states; (**a**–**c**) refer to States 0 to 2.

In order to facilitate the design the neural network, the size of the spectrogram needs to be converted into a square, where the length and width are equal. After that, the spectrograms are generated where the lengths and widths are each 512 pixels, respectively. Figure 3 shows the spectrograms for the different state of health, which varies with changes in frequency of the vibration of the bearings in time. Due to these changes, the spectrogram appears to be discontinuous since some signal components with higher frequencies vanish. The spectrogram of the vibration of the roller bearings is certainly a good feature to represent the state of health and the remaining useful life.

### 3.2. Construction of the Forward Propagation

**Figure 4:**
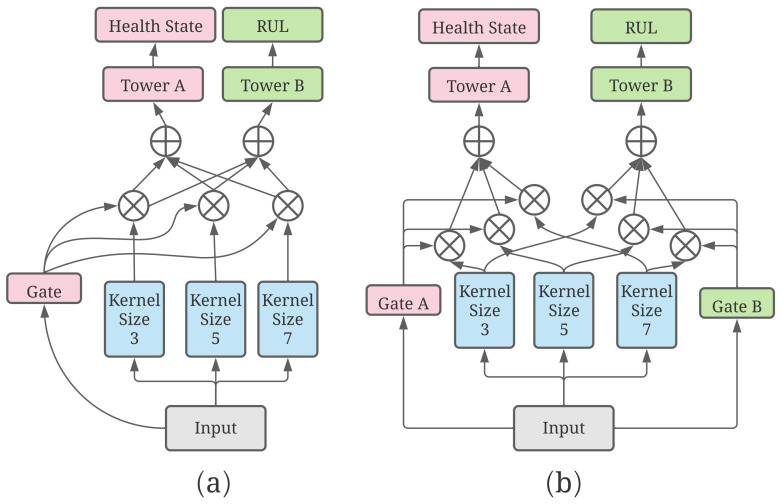
Construction of (**a**) proposed GMM-Net; (**b**) a multiscale net with two gates.

After converting the raw data to its spectrogram, a neural network shown in Figure 4 that encapsulates the essence of multiscale learning, multitask learning, and mixture of experts can be built. Subsequently, the spectrograms will be the inputs to the Gated Multiscale Multitask net (GMM-Net), which is perceived as the multiscale channels with different sizes of convolution kernels to obtain high level features. Furthermore, the gate network will give each high level feature of each multiscale channel a weight ranging between 0 and 1. These features are subsequently added to obtain a mixture of features, which is then given to the tower net in order to predict the state of health and the remaining useful life of the roller bearings. In addition, a similar network with two gates is shown to be compared.

#### 3.2.1. Multiscale Channels of Convolution Neural Network

Convolution neural networks are frequently used in different areas including pattern recognition, and it is widely used in fault diagnosis and remaining useful life prediction. The arithmetic operators of such neural networks include computations which involves convolution, pooling, flatten, linear regression, ReLU and softmax. Particularly, convolution *C* of a 2D input with size of i×j and a 2D kernel *K* with a size of m×n can be written mathematically as

(5) $$\begin{array}{c} C\left[i,j\right]=\sum_{m}\sum_{n}I\left[i-m,i-n\right]K\left[m,n\right] \end{array}$$

and the input can be observed in different scales by using kernels with different sizes. Intuitively, the bigger the kernel size is, the smaller the convolution. This means that the input is observed in a bigger scale to obtain the information globally. Then, the output becomes the input of the activation layer, which can be written mathematically as

(6) $$\begin{array}{c} O[i,j]=f(C[i,j]+b) \end{array}$$

where *b* is the bias, and *f* represents the activation function that is usually written as the ReLU function given as

(7) $$\begin{array}{c} ReLU(x)=max(x,0) \end{array}$$

**Figure 5:**
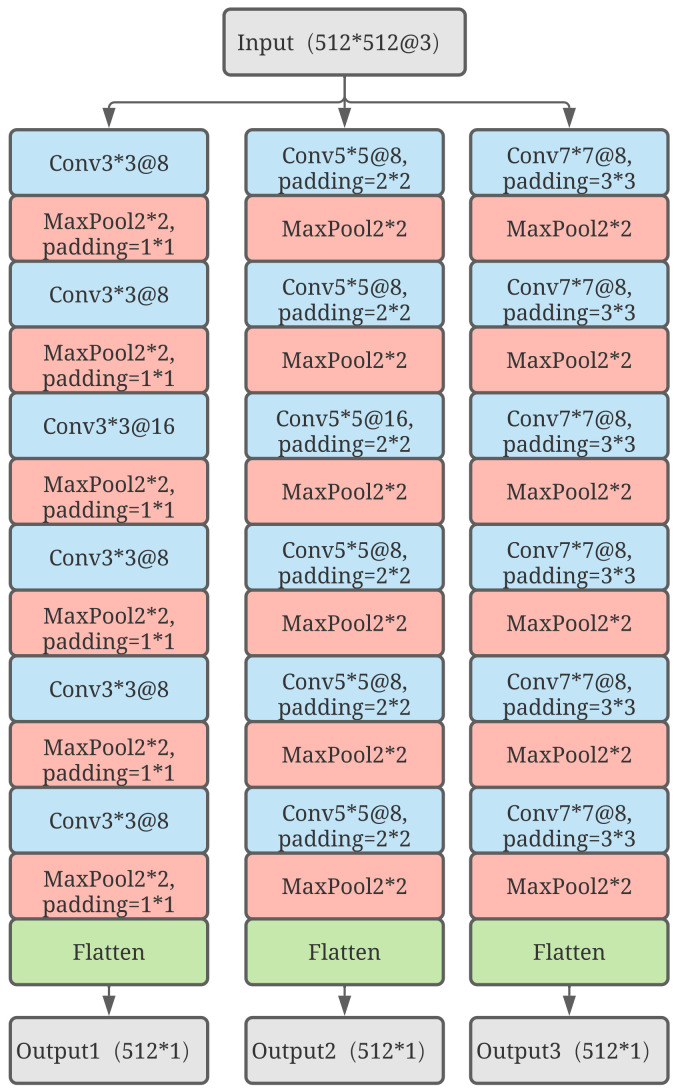
Details of the design of the three experts with different sizes of kernels.

Equation (7) introduces the nonlinearity into the network to improve the generalization ability of the network. Max pooling is used to reduce the size of the feature map and sensitivity of the output to the locations and spatially downsampling representations. When the pixels of the feature map reach a sufficiently small number, flatten computation can be used to reshape it into a one-dimensional tensor. Figure 5 shows the details of three channels with different sizes of kernels where the channels with different sizes of kernels have different perception fields and can observe the spectrogram in different scales to obtain a comprehensive result of the original input. In order to control the amount of parameters of the neural network model, the input and output channels of the layers have slight differences. In addition, an increase in the number of channels can reduce the influence of distortion by the downsampling operation.

#### 3.2.2. Gate Network and Tower Network

**Figure 6:**
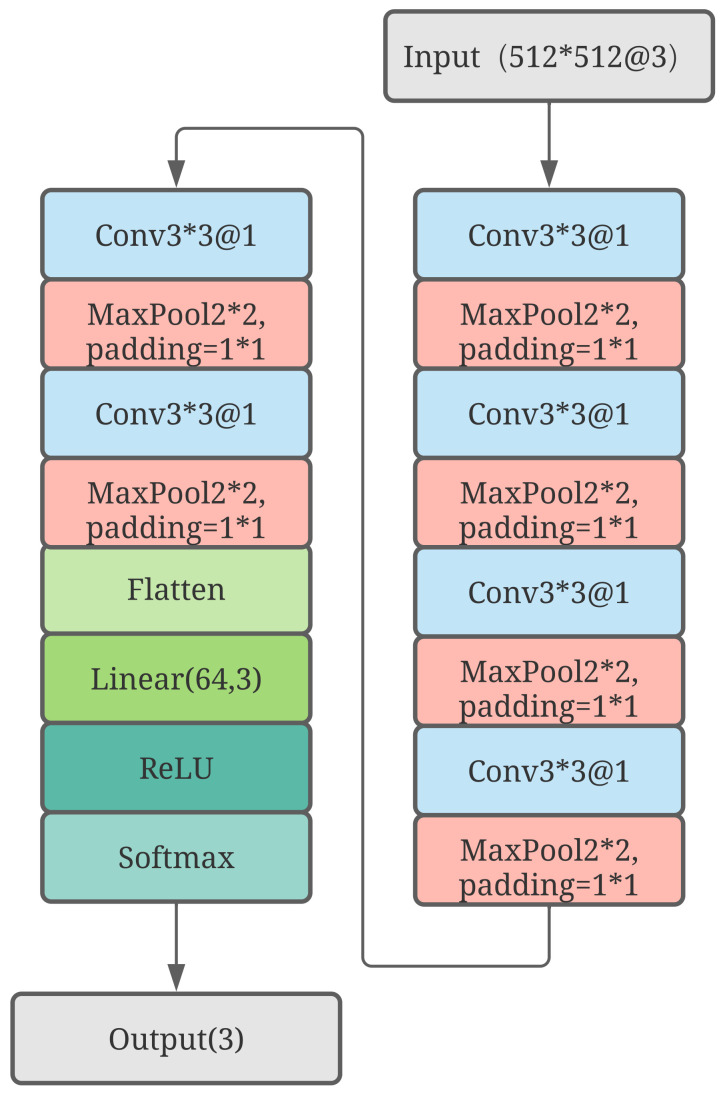
Details of the gate network.

The three channels with different kernel sizes can convert the input into three different high-level features in the same shape, but these three features need to be merged into one before passing it into the tower network to obtain the final prediction. Furthermore, features in different size of scales will need to be multiplied with different weights. Hence, the weights are dynamic, and this is the main reason why gate networks are designed to assign three different weights to multiply with different features. Figure 6 shows that a simpler network, which comprises less parameters using the three expert networks mentioned previously, is designed. The gate network downsamples the input and converts the input into an array which has the shape of 64 × 1. Linear regression is used to convert the array into three different values and the ReLU function is subsequently used to introduce the nonlinearity. Finally, the gate network uses the softmax function to obtain the three weights. These weights sum to one, which implies that these weights can be seen as weights that are used in weighted average. The softmax function of an *n*-dimensional input tensor can be mathematically described as

(8) $$Softmax\left(x_{i}\right)=\frac{exp(x_{i})}{\sum_{j}exp(x_{j})}$$

where $x_{i}$ represents one element of the input tensor. Equation (8) clearly shows that it can convert each of the three values into three other values that lie in the range from zero to one and whose sum is one. Since features in different scales have different weights, a gate network is designed to adapt the difference between the different scales. The parameters of the gate network can be trained so, after the model has been trained, the gate network can give the weights dynamically according to the input. The necessity of the gated network will be discussed in the next section. Following such an idea, we can also use two gates for two tasks just shown in Figure 6. For each task, a composite feature will be calculated by weighted average whose weights are given by the corresponding gate network, which can be represented as

(9) $$y=\sum_{i=1}^{n}g{\left(x\right)}_{i}f_{i}\left(x\right)$$

where $g{\left(x\right)}_{i}$ is the *i*th index of the output of g(x), representing the probability given to expert $f_{i}$ with a number *n*, and *g* represents a gate network that synthesizes the outputs from all experts. This equation also works with GMM-Net.

**Figure 7:**
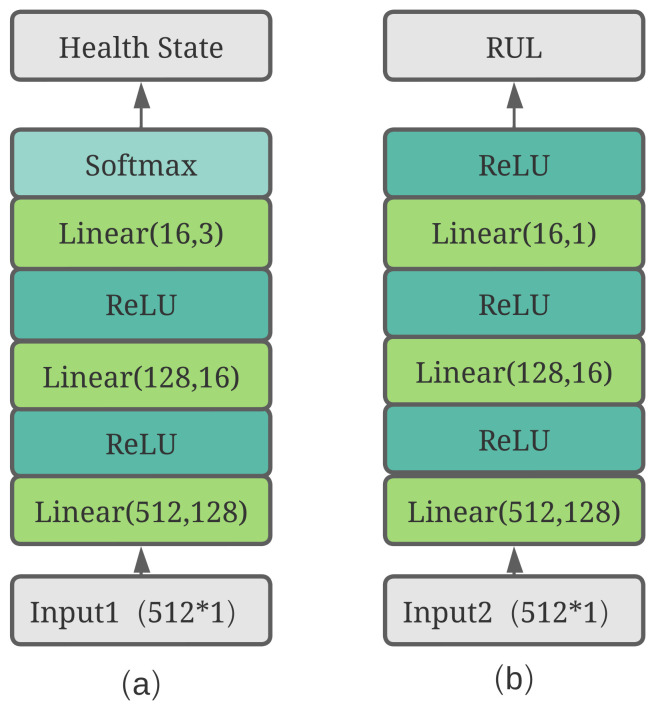
Details of two tower networks. (**a**) health state tower; (**b**) RUL tower.

Finally, the new composite features will be the inputs to the tower networks for each task. As shown in Figure 7, two different tower networks are used to compute the final results of the two tasks. There are slight differences between these two towers because the health state task is a classification problem that requires an array containing the possibilities of each state while the RUL prediction task is a regression problem that requires only a positive value.

### 3.3. Optimization of the Model

Usually, the loss of the prediction and the real value are needed to optimize the parameters of the neural network, which are defined as θ. For the remaining useful life estimation task, the loss function of the predicted value and the real value is the Mean Squared Error (MSE) loss function, which is widely used and can be described mathematically as

(10) $$\begin{array}{c} L_{1}\left(\theta \right)=\sum_{i=1}^{n}{\left(y_{pred}^{i}-y_{real}^{i}\right)}^{2} \end{array}$$

where number *i* represents the index of each predicted value $y_{pred}$ and the real value $y_{real}$. The cross entropy function is usually used in classification, and it is used in this paper for the health state judgement task, which can be described mathematically as

(11) $$\begin{array}{c} L_{2}\left(\theta \right)=\sum_{i=1}^{n}-log\frac{exp(x_{real}^{i})}{\sum_{j=1}^{C}exp(x_{j}^{i})} \end{array}$$

where it uses a format of negative logarithm of softmax function of the possibility of the right class that the network predicts. Different from works reported in [25], the equation of the total loss is described mathematically as

(12) $$\begin{array}{c} L_{Total}\left(\theta \right)=\omega L_{1}\left(\theta \right)+\left(1-\omega \right)L_{2}\left(\theta \right) \end{array}$$

where ω is the weight that ranges from zero to one. In comparison with [25], the authors made use of just one weight that ranges from zero to infinity. The proposed format in this paper also makes the search of the weight easier, and it has interpretability such that, when ω is zero, the total loss is just the cross-entropy loss where the training process is only concerned about the classification task. When ω increases, both tasks increase in importance, and the RUL prediction also increases in importance. However, when ω equals to one, the total loss is just the MSE loss, which implies that only RUL prediction is of importance.

If the loss is known a priori, the optimization can be calculated. In this paper, the Adam method [33] is used to optimize the parameter θ, which is different from the stochastic gradient descent method. The Adam method uses a moment vector which merges the information in the past moment and the present moment to obtain a proper estimation of the direction for the optimization to follow. Given the object function, the total loss function, f(θ), learning rate α, initial parameter vector $\theta_{0}$, and exponential decay rates $\beta_{1},\beta_{2}\in \left[0,1\right)$ can be initialized for the moment estimates, first moment vector $m_{0}=0$, second moment vector $v_{0}=0$, and time step t=0. When $\theta_{t}$ does not become converged, *t* should increase and be used to compute the gradient of the object function at time step *t* to obtain first-hand information similar to the stochastic gradient descent method, which can be described as

(13) $$\begin{array}{c} g_{t}={▽}_{t}f_{t}\left(\theta_{t-1}\right) \end{array}$$

Then, the biased first moment estimate and the biased second raw moment estimate can be updated similar to the momentum method, which can be described as

(14) $$\begin{array}{ccc} m_{t} & = & \beta_{1}m_{t-1}+\left(1-\beta_{1}\right)g_{t} \end{array}$$

(15) $$\begin{array}{ccc} v_{t} & = & \beta_{2}v_{t-1}+\left(1-\beta_{2}\right)g_{t}^{2} \end{array}$$

where $g_{t}^{2}$ refers to the element-wise square $g_{t}⊙g_{t}$. These two computations can estimate the information in the next time step, which can speed up the optimization. Then, the bias-corrected first moment estimate and the bias-corrected second raw moment estimate are obtained in order to make the estimates more proper. These two computations can be described in the following equations as

(16) $$\begin{array}{ccc} \hat{m_{t}} & = & \frac{m_{t}}{1-\beta_{1}^{t}} \end{array}$$

(17) $$\begin{array}{ccc} \hat{v_{t}} & = & \frac{v_{t}}{1-\beta_{2}^{t}} \end{array}$$

where $\beta_{1}^{t},\beta_{2}^{t}$ represents values that donate $\beta_{1},\beta_{2}$ to the power *t*. Finally, $\theta_{t}$ can be computed using the following equation:

(18) $$\begin{array}{c} \theta_{t}=\theta_{t-1}-\alpha \frac{\hat{m_{t}}}{\sqrt{\hat{v_{t}}}+\epsilon } \end{array}$$

to update the parameter, and ϵ is set to a value slightly greater than zero to avoid $\hat{m_{t}}$ being divided by zero. Therefore, the above explains how the network parameters are trained.

## 4. Experiments and Discussion

### 4.1. Data Description and Arrangement

**Figure 8:**
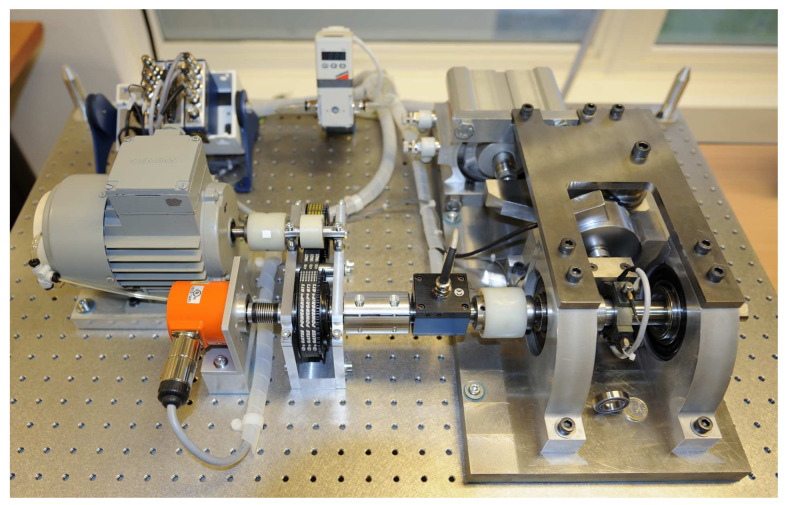
Overview of the PRONOSITIA [34].

Data used for validation of our proposed method were taken from the IEEE PHM 2012 Prognostic challenge, which were provided by FEMTO-ST Institute [34]. This dataset is open-source and is commonly used in RUL prediction, which means that the performance of our proposed method can be validated and compared more easily. As shown in Figure 8, PRONOSTIA is composed of three main parts: a rotating part, a degradation generation part, and a measurement part. The PRONOSTIA platform conducts run-to failure experiments and tests were stopped when the amplitude of vibration signal exceeded 20 g. Bearing1_1 dataset and Bearing1_3 dataset including 2803 and 2375 segments of the vibration signals are chosen. The operating conditions for the chosen data are such that the rotation speed was 1800 rpm, and the load was 4000 N. The sampling frequency is 25.6 kHz, and each segment has 2560 samples recorded in 0.1 s each 10 s, which implies the shape of the raw input is 2560 × 1.

First, the raw input needs to be converted into the spectrogram with a shape of 512 × 512 × 3 similar to Figure 3. Since these raw data have no labels such as RUL and health state, they will need to be labeled accordingly. The RUL is computed following the equation as follows:

(19) $$\begin{array}{c} RUL\left(t\right)=\left\{\begin{array}{cc} 1 & 0\leq t\leq t_{start}\\ 1-\frac{t-t_{start}}{t_{end}-t_{start}} & t_{start}\leq t\leq t_{end}\\ 0 & t\geq t_{end} \end{array}\right. \end{array}$$

where $t_{start}$ is the start point of the degradation, $t_{end}$ is the end of the degradation when the rolling bearings are broken. The degradation is assumed to be piecewise linear, which means that the degradation does not occur when the rolling bearings are working until the rms values of the raw data are over the threshold i.e., mean (rms) ± (6 × σ) where the mean value of the rms before the degradation begins [35]. The rms values between the segment with index from 500 to 1000 are chosen to compute the mean(rms) for dataset 1_1. The rms values between the segment with index from 100 to 700 are chosen to compute the mean(rms) for dataset 1_3. When the rms of one sample is more than the threshold, the index of this sample is considered as the start point of degradation.

**Figure 9:**
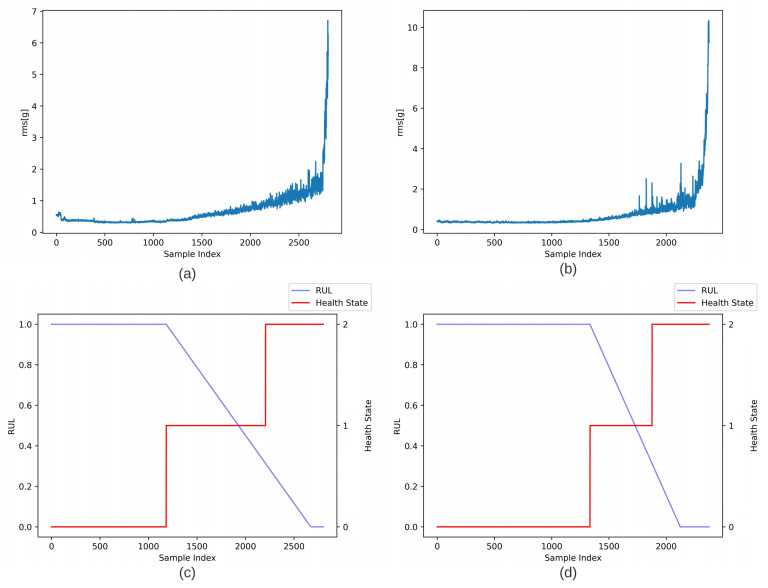
(**a**,**b**) RMS of each samples of dataset 1_1 and 1_3; (**c**,**d**) RUL and health state of dataset 1_1 and 1_3.

The end of degradation is when the rms value of a segment is more than 2 g. With these two points, the RUL of each segment can be labeled. For health state label, the dataset can be divided by finding the turning point when the rms value of a segment is more than 1.2 g along with the start point and the end point of the degradation. Thus, with the start point and turn point, the dataset can be divided into three classes from state 0 to 2. Similar to Figure 9, 2803 samples in dataset 1_1 and 2375 samples in dataset 1_3 were labeled with the responding RUL and health state labels.

### 4.2. Results and Discussion

After labeling the data, each dataset can be randomly divided into two parts, which includes the train dataset with 1962 and 1662 samples that covers about 70 percent of each dataset and the test dataset with 841 and 713 samples that cover the remaining 30 percent. The proposed GMM-net, a multiscale network with two gates and a sole multiscale network, has been implemented in Pytorch 1.8 software system built on Python 3.7.4. The hardware used includes the GPU NVIDIA GTX 1650 SUPER and CPU Intel i7-10700. All three models have been trained with 20 epochs.

**Figure 10:**
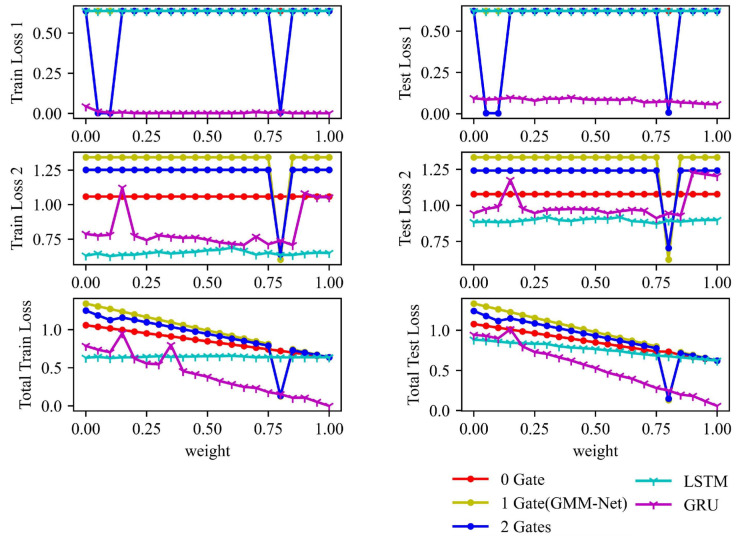
Final train and test losses of five models by changing the weight ω.

Frequently used LSTM [24] and GRU [8] are also implemented to be compared with our method. The final three losses results including MSE loss(loss 1), cross-entropy loss (loss 2) and total loss are presented in Figure 10. The weight ω has been changed in steps of 0.05 from zero to one. It is observed that, when ω=0.8, GMM-Net has a better result in both losses. The model with no gate is stuck in a value, and the model with two gates has worse losses. These shows the effectiveness of GMM-Net in dealing with the features in three different scales.

**Figure 11:**
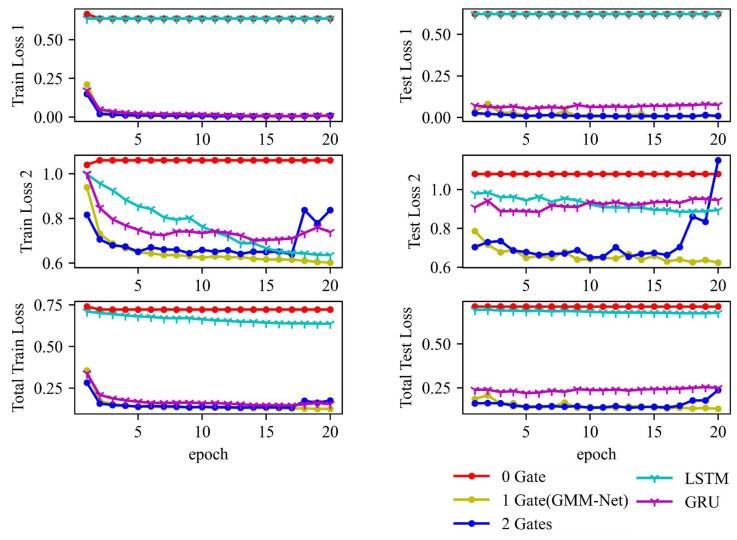
Loss trends of five models.

From Figure 11, it is observed that the losses obtained from the models converge when epoch number increases. In particular, MSE loss and cross-entropy loss are of interest, and it is clear that GMM-Net can converge eventually, while the model with no gating network fails to converge, which can prove that the gating network can deal with the differences between different scales properly but more gating networks can give more random weights to two tower networks that make the estimation of health state and RUL worse. The proposed GMM-Net can achieve an average train loss 1 of 0.006 and train loss 2 of 0.602, while the test loss 1 is 0.007, and the test loss 2 is 0.624. LSTM and GRU also achieve higher losses than GMM-Net, which can also show the goodness of GMM-Net.

**Figure 12:**
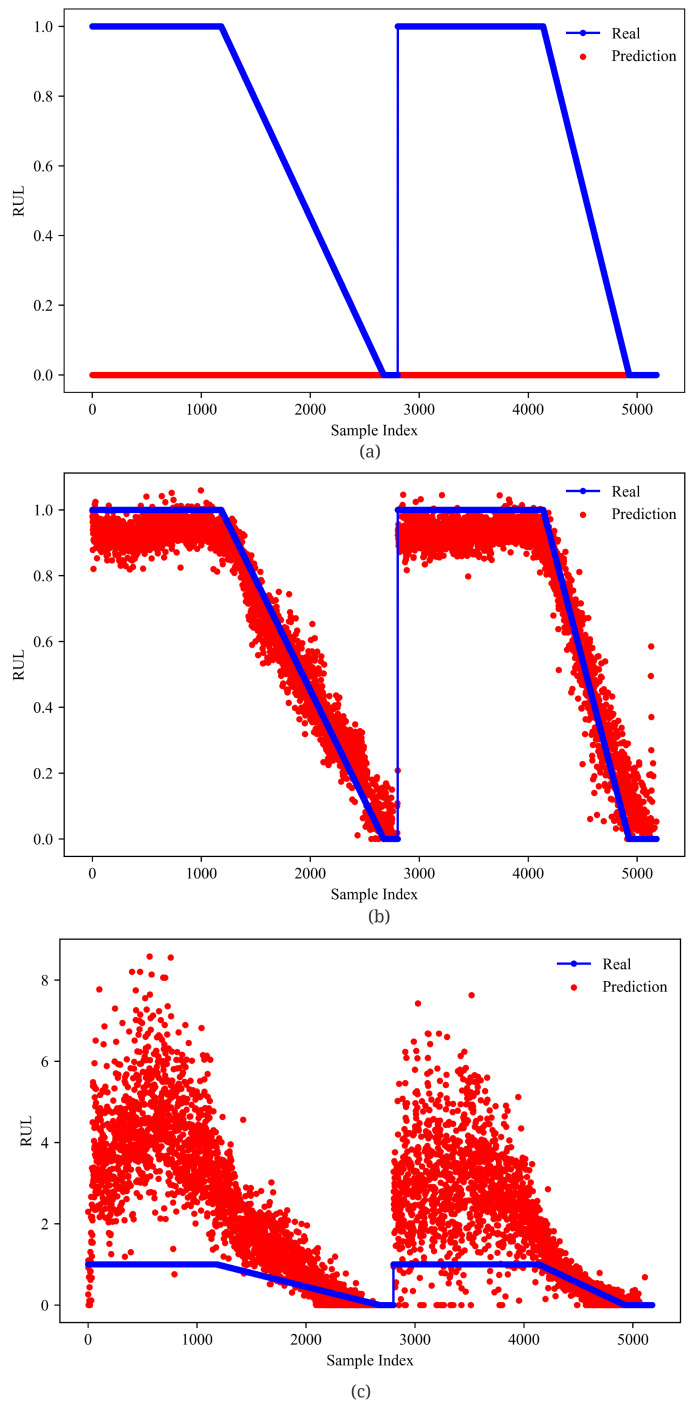
RUL prediction of models (**a**) 0 Gate. (**b**) 1 Gate(GMM-Net); (**c**) 2 Gates. Samples from bearing 1_1 and bearing 1_3 are joint from left to right.

**Figure 13:**
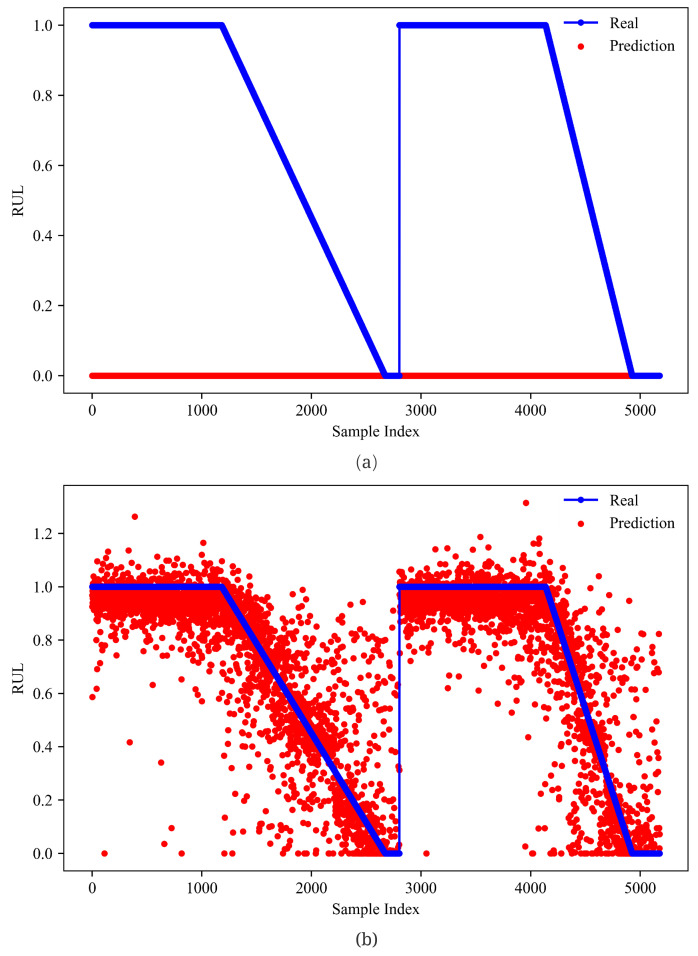
RUL prediction of models (**a**) LSTM. (**b**) GRU. Samples from bearing 1_1 and bearing 1_3 are joint from left to right.

**Figure 14:**
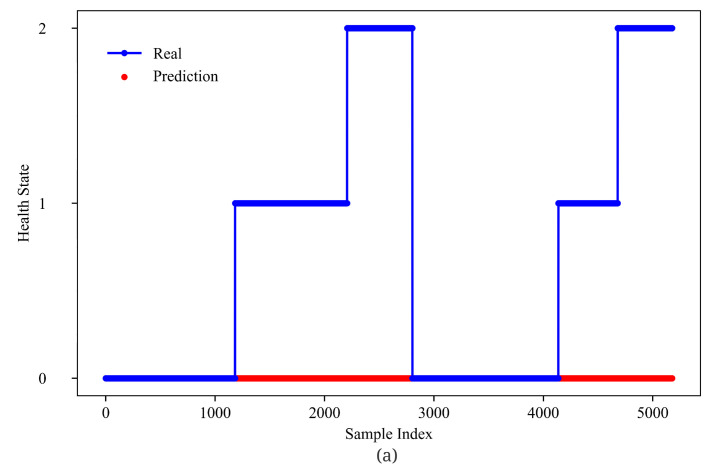

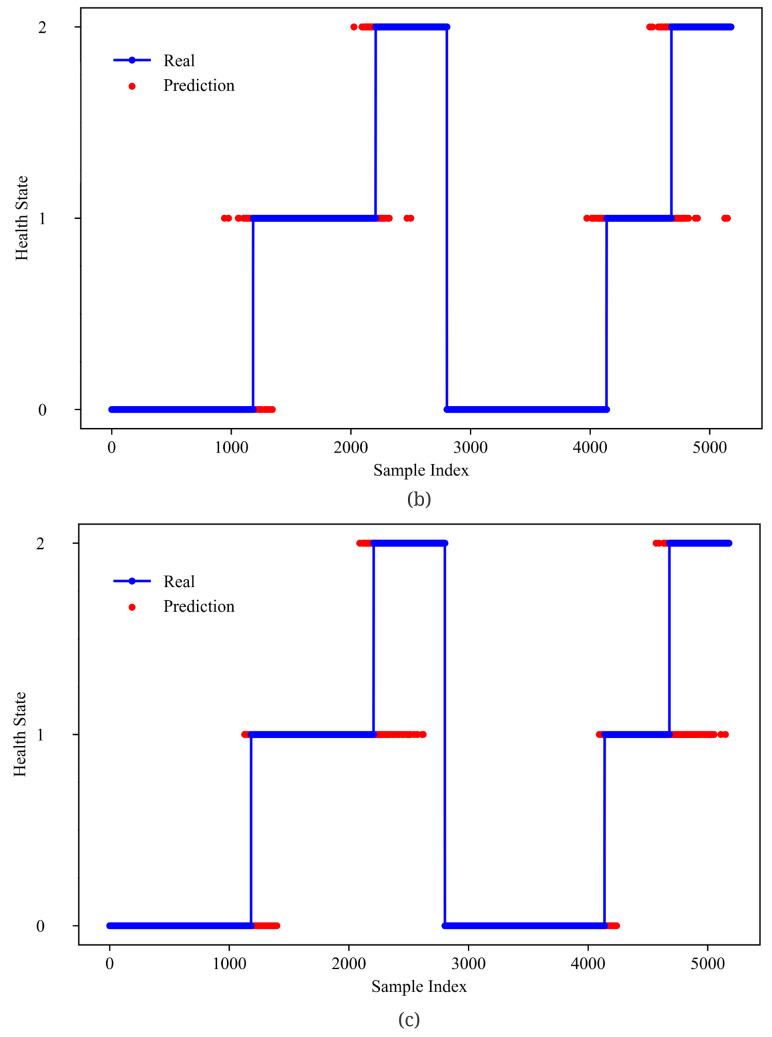
Health state prediction of five models (**a**) 0 Gate; (**b**) 1 Gate(GMM-Net); (**c**) 2 Gates. Samples from bearing 1_1 and bearing 1_3 are joint from left to right.

**Figure 15:**
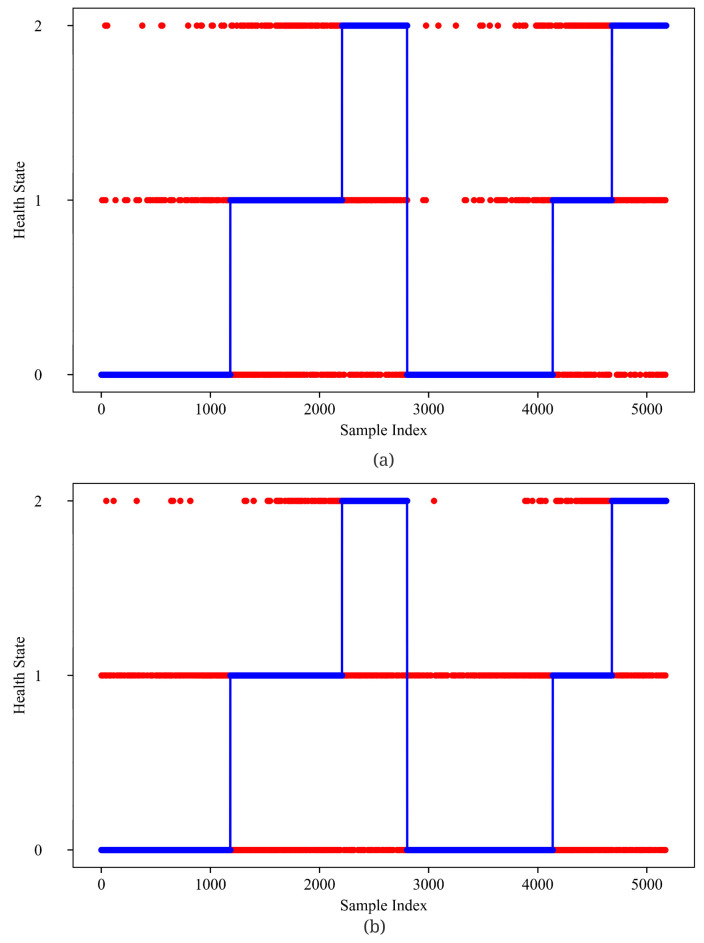
Health state prediction of five models (**a**) LSTM; (**b**) GRU. Samples from bearing 1_1 and bearing 1_3 are joint from left to right.

From Figure 12 and Figure 13, GMM-Net has a smaller MSE loss with RUL prediction, which makes the predicted value closer to the real ones than LSTM and GRU. From Figure 14 and Figure 15, it is observed that GMM-Net also does better in the classification task with an overall accuracy of 94.84%. Thus, both tasks can be carried out by GMM-Net with a smaller loss. Hence, the GMM-Net can be a proper model for classification and regression.

To evaluate our GMM-Net further, the average RUL loss and the accuracy of all three health states are calculated. As given in Table 1, although GMM-Net achieves an accuracy of 96.54% in state 0, GMM-Net does best in the rest. In detail, GMM-Net achieves an MSE loss of 0.00586 in state 0, 0.00522 in state 1, and 0.00760 in state 2. The accuracy of the prediction of the health state is 96.54% in state 0, 93.10% in state 1, and 93.42% in state 2. Although the model with no gate and two gate has a better accuracy in state 0, they do worse in the rest. For example, the model with no gate fails to predict the health state when state is 1 or 2. The model with two gates fails to predict the RUL properly in States 1 and 2. This is the same with LSTM and GRU models. The LSTM achieves an accuracy of 93.33% in health State 0, an accuracy of 76.37% in health state 1, and an accuracy of 72.94% in health State 2. However, the LSTM fails to predict the RUL properly. The GRU achieves an accuracy of 83.48% in health State 0, an accuracy of 60.28% in health State 1 and an accuracy of 75.86% in health State 2. The GRU does better in predicting RUL than the LSTM, but the average MSE value of RUL is more than the GMM-Net.

Besides the MSE and the accuracy to prove the goodness of GMM-Net, it is necessary to prove that our method can be used in the real-time environment. From the Table 2, it is shown that the training time per epoch is 61 s for model with no gate. When the number of gates increases, the training time increases to 70 s and 84 s. For LSTM and GRU, it costs more training time per epoch, which are 130 s and 98 s. The differences among the training time can be explained by the model size. For the models with 0, 1, and 2 gates, the model size is 685 kB, 693 kB, and 702 kB. However, LSTM and GRU have a model size of 420,546 kB and 318,105 kB, which implies that it will cost much more memory space to deploy them to the hardware.

## 5. Conclusions

In this paper, a model for health assessment and remaining useful life prediction is proposed that incorporates time-frequency domain representation, multiscale learning, multitask learning, and the mixture of experts. The GMM-Net is proposed, which have been verified by our experiments and discussions. It can have a good performance in remaining useful life prediction and a better performance in classification of health state. The STFT is used to convert the raw data into the spectrograms to make them more interpretable. Furthermore, a neural network with three expert nets and a lightweight gating net for two tasks that can dynamically assign weights for three features in different scales are built. It is observed that, when the model has no gating net, the performance is not so good, and the losses are stuck at a value; hence, more work will be needed to address this issue in the future work. The training time and the model size are further discussed to prove the goodness of the proposed method.

## Figures and Tables

**Table 1:** Average MSE and accuracy of three health states.

Methods	Overall		Health State 0		Health State 1		Health State 2	
	Average MSE	Accuracy (%)	Average MSE	Accuracy (%)	Average MSE	Accuracy (%)	Average MSE	Accuracy (%)
LSTM [24]	0.633	83.89	1.0	93.33	0.470	76.37	0.0212	72.94
GRU [8]	0.02955	74.86	0.0130	83.48	0.0424	60.28	0.0492	75.86
0 Gate (GMM-Net)	0.632	48.63	1.0	100.0	0.470	0	0.0212	0
2 Gates (GMM-Net)	3.937	89.03	7.585	99.40	0.811	85.50	0.0162	70.20
1 Gate (GMM-Net)	0.00604	94.84	0.00586	96.54	0.00522	93.10	0.00760	93.42

**Table 2:** Train time and model size of models.

Methods	Training Time per Epoch (s)	Model Size (KB)
LSTM [24]	130	420,546
GRU [8]	98	318,105
0 Gate (GMM-Net)	61	685
1 Gate (GMM-Net)	70	693
2 Gates (GMM-Net)	84	702

## Data Availability

No new data were created. Data can be found through our paper in Section 4.
